# Application of Fluorescence- and Bioluminescence-Based Biosensors in Cancer Drug Discovery

**DOI:** 10.3390/bios14120570

**Published:** 2024-11-24

**Authors:** Tynan Kelly, Xiaolong Yang

**Affiliations:** Department of Pathology and Molecular Medicine, Queen’s University, Kingston, ON K7L 3N6, Canada; 18tnpk@queensu.ca

**Keywords:** biosensor, cancer, drug discovery, high-throughput screening, FRET, TR-FRET, BRET, NanoBRET, bioluminescence, fluorescence

## Abstract

Recent advances in drug discovery have established biosensors as indispensable tools, particularly valued for their precision, sensitivity, and real-time monitoring capabilities. The review begins with a brief overview of cancer drug discovery, underscoring the pivotal role of biosensors in advancing cancer research. Various types of biosensors employed in cancer drug discovery are then explored, with particular emphasis on fluorescence- and bioluminescence-based technologies such as FRET, TR-FRET, BRET, NanoBRET, and NanoBiT. These biosensors have enabled breakthrough discoveries, including the identification of Celastrol as a novel YAP-TEAD inhibitor through NanoBiT-based screening, and the development of TR-FRET assays that successfully identified Ro-31-8220 as a SMAD4R361H/SMAD3 interaction inducer. The integration of biosensors in high throughput screening and validation for cancer drug compounds is examined, highlighting successful applications such as the development of LATS biosensors that revealed VEGFR as an upstream regulator of the Hippo signaling pathway. Real-time monitoring of cellular responses through biosensors has yielded invaluable insights into cancer cell signaling pathways, as demonstrated by NanoBRET assays detecting RAF dimerization and HiBiT systems monitoring protein degradation dynamics. The review addresses challenges linked to biosensor applications, such as maintaining stability in complex tumor microenvironments and achieving consistent sensitivity in HTS applications. Emerging trends are discussed, including integrating artificial intelligence and advanced nanomaterials for enhanced biosensor performance. In conclusion, this review offers a comprehensive analysis of fluorescence- and bioluminescence-based biosensor applications in the dynamic cancer drug discovery field, presenting quantitative evidence of their impact and highlighting their potential to revolutionize targeted cancer treatments.

## 1. Introduction

### 1.1. Brief Overview of Cancer Drug Discovery

The field of cancer drug discovery has undergone transformative advancements in recent years, driven by technological innovations and a deeper understanding of molecular cancer biology. This progress spans from improvements in hight throughput screening (HTS) and small molecule (SM) therapies to new developments in artificial intelligence (AI) applications and targeted therapies. The integration of SMs into targeted cancer therapies, phenotypic screenings, and structural biology has significantly expanded the scope and efficacy of cancer treatments [1,2,3].

HTS remains a cornerstone of drug discovery, enabling the rapid evaluation of thousands of compounds. Researchers employ various HTS methods, including label-free assays [e.g., surface plasmon resonance (SPR), Biocore, isothermal titration calorimetry (ITC)], fluorescence-based assays [e.g., fluorescence polarization (FP), anisotropy, fluorescent resonance energy transfer (FRET), time-resolved FRET (TR-FRET), and fluorescence lifetime analysis], bioluminescent based assays [e.g., NanoBiT (NanoLuc Binary Technology), NanoBRET (NanoLuc BRET), AlphaScreen, and luciferase reporter), binding based assays [e.g., proteolysis targeting chimera (PROTAC), covalent drug, mass-spec technology, and DNA encoding library (DEL)], and cell-based assays [4,5,6]. While traditional HTS relied on two-dimensional (2D) cultures, which often fell short of mimicking the complex tumor microenvironment, recent developments incorporate 3D multicellular spheroids or animal models that offer more physiologically relevant models for studying drug efficacy and resistance [7]. These improved models significantly enhance the ability to predict therapeutic outcomes [5,7,8], particularly when targeting cancer cells within their unique microenvironment.

SMs in targeted cancer therapies represent one of the most significant advancements in oncology over the past two decades. Due to their small size, these compounds can penetrate cells and inhibit intracellular signaling pathways, offering advantages over monoclonal antibodies, which typically act on extracellular targets [3,9]. Another advantage of SMs is that they can be administered orally, unlike other modalities. The Food and Drug Administration (FDA) has approved more than 43 SM inhibitors for oncology applications, with many of these drugs exhibiting fewer side effects and higher efficacy compared to traditional cytotoxic chemotherapies [3,10]. Examples of successful targeted therapies include selective kinase inhibitors, such as sorafenib and sunitinib, which target multiple kinases across various cancer types. This class of drugs has evolved to include selective inhibitors that focus on specific components of cancer signaling pathways for more personalized treatment strategies based on individual tumor genetics [3,9]. Selective epithelial growth factor receptor (EGFR) inhibitors like erlotinib and gefitinib have revolutionized the treatment of non-small cell lung cancer (NSCLC), particularly in patients with EGFR mutations. Similarly, BRAF inhibitors such as vemurafenib have shown efficacy in melanoma patients with BRAF V600E mutations, further emphasizing the role of genomic markers in guiding therapy [11].

One of the ongoing challenges in cancer drug discovery is overcoming drug resistance, which often arises through mechanisms such as secondary mutations in target proteins, the activation of compensatory pathways, or drug efflux [1,2,3]. Researchers are addressing this issue by developing next-generation inhibitors that target resistance mechanisms and by exploring combination therapies that prevent cancer cells from evading the effects of single-agent treatments [9]. Additionally, phenotypic screening and pooled Clustered Regularly Interspaced Short Palindromic Repeats (CRISPR) approaches are increasingly being used to uncover new targets for cancer development. These strategies allow for the identification of compounds that modulate cancer cell behavior based on observable traits rather than predefined molecular targets [6,11]. CRISPR-based screens have been critical in identifying genes that contribute to drug resistance, providing valuable insights into potential therapeutic targets [6].

AI has also significantly impacted cancer drug discovery, particularly in protein structure prediction. AI-powered tools, such as AlphaFold2, have dramatically improved the accuracy of protein structure models, which are crucial for rational drug design. This advancement has accelerated the discovery of new SM inhibitors by enabling researchers to target previously “undruggable” proteins with greater precision [2]. AI-driven structural predictions are helping to identify new binding sites on oncogenic proteins, offering fresh avenues for therapeutic intervention. Additionally, the drugging of “undruggable” targets, including key oncogenes like RAS and MYC, has advanced through approaches such as PROTAC. PROTACs function by tagging disease-causing proteins for degradation rather than simply inhibiting them, providing a new modality for addressing proteins that were previously difficult to target with traditional SMs [12].

The integration of molecular screening techniques such as next-generation sequencing (NGS) and connectivity mapping has further enhanced the drug discovery process. These techniques allow researchers to match patients with therapies based on their unique molecular profiles, ushering in an era of precision medicine. Specifically, connectivity mapping has accelerated the discovery of effective treatments by linking transcriptomic data with existing drug compounds, leading to novel uses for established drugs [3,6]. The repurposing of existing drugs for new cancer indications, guided by these tools, has reduced the time and cost associated with developing new therapies [11].

In conclusion, the field of cancer drug discovery is experiencing rapid advancements driven by the convergence of HTS, AI-driven insights, and novel SM therapies. These developments are pushing the boundaries of what is possible, allowing for more targeted, effective, and personalized cancer treatments. Despite ongoing challenges like drug resistance and the complexity of cancer biology such as heterogenicity, the collective progress highlighted in these studies offers a promising foundation for future breakthroughs in oncology.

### 1.2. Significance of Biosensors in Cancer Research

Biosensors have revolutionized cancer research by offering high sensitivity, specificity, and real-time monitoring capabilities for protein levels and structure and protein–protein interactions (PPIs), significantly advancing the fields of diagnostics, therapeutic monitoring, and drug discovery [13,14,15]. These technologies have provided invaluable tools for early cancer detection, particularly in cases where traditional diagnostic methods fall short due to complexity, invasiveness, or cost [16,17,18,19]. One of the biosensors’ most significant contributions to cancer research lies in early-stage diagnosis, particularly through the detection of circulating tumor biomarkers. Researchers can detect biomarkers such as circulating tumor cells (CTCs), circulating tumor DNA (ctDNA), and various proteins with high precision. For example, studies have demonstrated that label-free electrochemical biosensors effectively detect these biomarkers in body fluids like blood, offering non-invasive methods for early cancer detection [16,18]. These approaches eliminate the need for complex sample preparation and labeling, making them more accessible and rapid for clinical applications. For example, these biosensors enable breast cancer diagnosis by identifying key biomarkers such as HER2, CA15-3, and circulating microRNAs (miRNAs, e.g., miR-21 and miR-155) [16,17].

Moreover, biosensors have transformed the landscape of drug discovery and therapeutic monitoring in cancer research [20,21]. High-throughput biosensor assays, including split-luciferase complementation assays (SLCA), play crucial roles in screening potential drug candidates by measuring PPIs and receptor activity in cancer cells. These biosensors enable researchers to identify compounds that modulate oncogenic signaling pathways, such as the Hippo or Wnt pathways, which are central to cancer cell proliferation and survival [22,23,24,25]. The use of these advanced sensor technologies helps accelerate the identification and optimization of SM inhibitors, significantly shortening the drug discovery process. In addition, biosensors are being used for their role in personalized cancer therapy. With their ability to measure real-time responses to drugs, biosensors allow for precise monitoring of therapeutic efficacy, enabling adjustments in treatment regimens based on how a patient’s cancer cells respond. This approach is especially promising in targeted therapies, where biosensors can help identify resistance mechanisms, such as mutations in ctDNA or protein alterations, allowing clinicians to tailor treatments to individual patients [17,18].

In summary, biosensors have become integral to the advancement of cancer research, particularly in the realms of early detection and personalized medicine. By providing highly sensitive, non-invasive, and real-time diagnostic tools, biosensors enhance our ability to detect cancer earlier, monitor disease progression more accurately, and optimize therapeutic interventions. As biosensor technology continues to evolve, it promises to further revolutionize the way cancer is diagnosed, treated, and monitored, ultimately improving patient outcomes across various cancer types.

## 2. Types of Fluorescence- and Bioluminescence-Based Biosensors

### 2.1. Fluorescence Biosensors

Fluorescence biosensors operate through the excitation of fluorescent molecules (fluorophores) by an external light source, followed by light emission at a different wavelength. These biosensors effectively detect biomolecular interactions, conformational changes, and enzyme activities.

**Figure 1:**
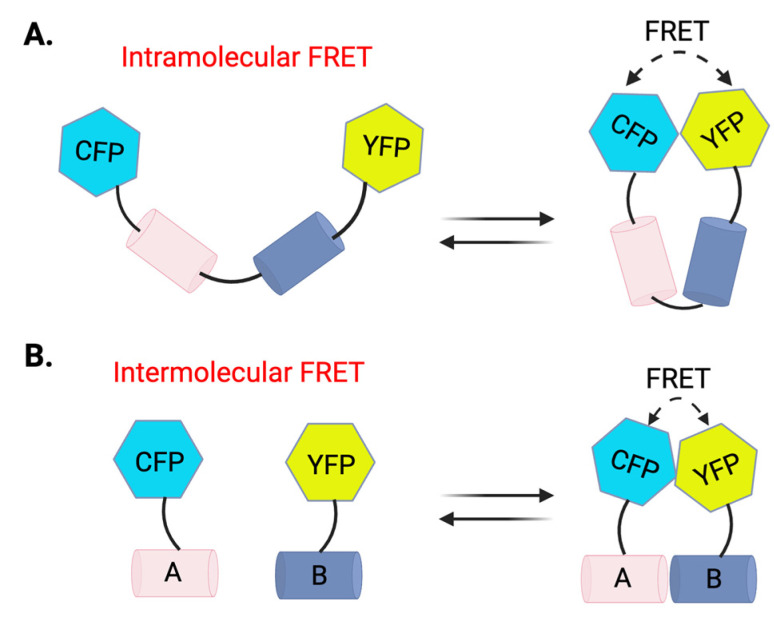
Schematic representation of intramolecular and intermolecular FRET biosensors. (**A**) Intramolecular FRET. A single protein with CFP and YFP attached at two ends undergoes conformational changes, bringing CFP and YFP into proximity. Energy transfer occurs when CFP is excited, leading to emission from YFP. (**B**) Intermolecular FRET. Two interacting proteins, A and B, are tagged with CFP and YFP, respectively. When the proteins interact closely, FRET occurs as energy from the donor (CFP) is transferred to the acceptor (YFP), resulting in fluorescence from YFP. These FRET configurations allow the monitoring of protein interactions and conformational changes in live cells.

Förster resonance energy transfer (FRET) is a distance-dependent energy transfer process between two light-sensitive molecules, typically called the “donor” and “acceptor”. When the donor fluorophore is excited by an external light source, it can transfer its energy non-radiatively to an acceptor fluorophore if they are within 1–10 nanometers of each other. The efficiency of FRET is highly sensitive to the distance between the donor and acceptor fluorophores, making it an excellent method for detecting molecular interactions and conformational changes in real time. In a typical fluorescence biosensor system, the donor (e.g., cyan fluorescent protein (CFP)) emits light only when it is in close proximity to the acceptor [e.g., yellow fluorescent protein (YFP)], which then re-emits light at a different wavelength. This setup enables the detection of dynamic processes, such as protein–protein interactions, protein-small molecule interactions, or changes in protein conformation within live cells (Figure 1). Intramolecular FRET and intermolecular FRET biosensors (Figure 1) are particularly valuable for studying dynamic changes within individual proteins and interactions between proteins. The energy transfer efficiency between the donor and acceptor depends on their proximity, typically within 1–10 nanometers.

**Figure 2:**
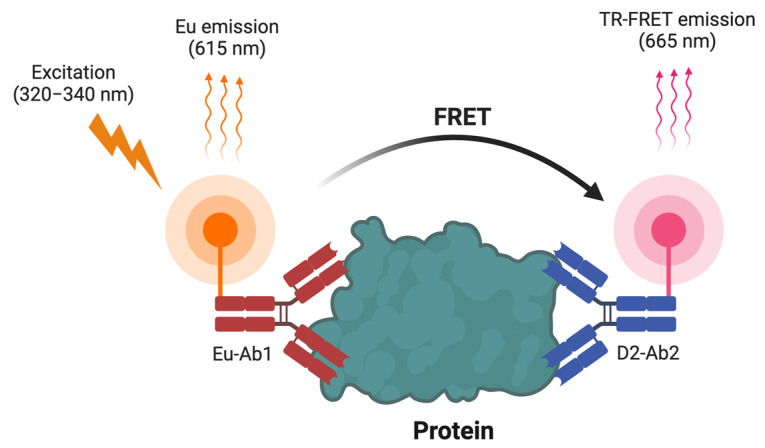
Schematic presentation of TR-FRET biosensors. The target protein is bound by two antibodies: Eu-Ab1, labeled with a europium (Eu) donor fluorophore, and D2-Ab2, labeled with the D2 acceptor fluorophore. Upon excitation at 320–340 nm, the Eu donor emits at 615 nm. When the antibodies are in close proximity due to binding the same protein or two interacting proteins, FRET occurs from the Eu donor to the acceptor, resulting in a TR-FRET emission at 665 nm.

Time-Resolved FRET (TR-FRET) represents an advanced application of FRET technology [26,27]. It is different from FRET in three aspects: (1) Fluorophores: FRET uses traditional fluorophores, while TR-FRET uses lanthanide-based fluorophores, such as terbium or europium as the donor fluorophore, which has a long light emission lifetime at approximately 615 nm wavelength after excitation at 320–340 nm (Figure 2); (2) Measurement techniques: FRET measures emission instantly, while TR-FRET uses a time delay (typically 50–100 microseconds) in detection, which significantly reduces short-lived autofluorescence noise (Figure 2) and improves the signal-to-noise ratio; (3) Sensitivity: TR-FRET generally offers a high-sensitivity, making TR-FRET ideal for HTS applications in drug discovery where sensitivity is critical. In TR-FRET biosensors, when the donor and acceptor are brought into proximity through molecular interactions (either intramolecularly in a protein or intermolecularly between two proteins), energy is transferred to the acceptor, causing it to emit at a characteristic wavelength (665 nm; Figure 2).

Both FRET and TR-FRET fluorescent biosensors are used extensively to monitor intracellular events, such as calcium signaling, kinase activity, and protein folding, providing valuable insights into cellular processes at a molecular level [27,28,29]. Fluorescence biosensors also offer high temporal resolution, making them suitable for applications where precise real-time tracking of fast cellular events is required. For example, green fluorescent protein (GFP) and its variants have been engineered into biosensors to visualize the localization and activity of proteins within living cells. The real-time imaging capabilities of fluorescence biosensors have been instrumental in studying cancer cell behavior, drug responses, and the dynamics of various oncogenic signaling pathways [30]. Fluorescence biosensors are widely used in HTS to identify potential drug candidates by detecting changes in fluorescent signals corresponding to the binding or inhibition of target proteins. This is particularly important in cancer drug discovery, where rapid screening of large chemical libraries is essential for identifying new therapeutic molecules.

**Figure 3:**
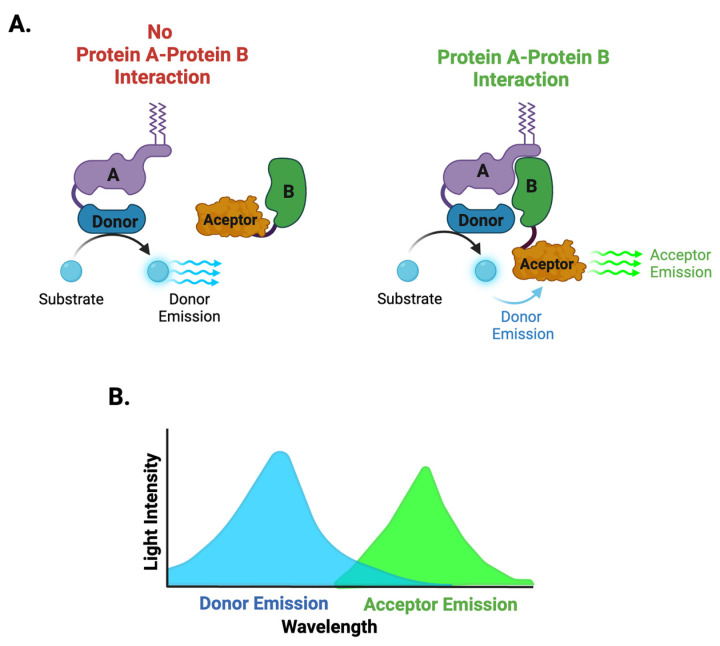
Schematic representation of the BRET and NanoBRET biosensor. (**A**) The energy donor such as a luciferase enzyme (RLuc or NanoLuc) is fused to one protein of interest (Protein A), while a fluorescent acceptor is attached to the interacting partner (Protein B). When Protein A and B interact within a close distance (~5–10 nm), upon substrate (e.g., coelenteramide) oxidation, the luciferase enzyme (donor) releases energy in the form of photons, with specific emission wavelengths (e.g., ~480 nm for RLuc). The acceptor fluorophore absorbs the donor emission and re-emits it at a higher wavelength (e.g., ~530 nm for YFP), producing a measurable light signal (**B**) when protein–protein interactions bring the donor and acceptor close together.

Bioluminescence Resonance Energy Transfer (BRET) and its enhanced version, NanoBRET, are powerful techniques used to study molecular interactions in live cells. BRET is a luciferase-based biosensing technique that eliminates the need for external excitation, which helps to reduce background noise. In BRET, a luciferase enzyme [often Renilla luciferase (RLuc)] serves as the energy donor, and a fluorescent acceptor, such as YFP, receives the energy. Upon oxidation of a substrate (e.g., coelenterazine), the luciferase enzyme emits light at a specific wavelength (typically around 480 nm). If the acceptor fluorophore is close enough (approximately 1–10 nm), it absorbs this emitted energy and re-emits it at a longer wavelength (e.g., 530 nm; Figure 3). NanoBRET is an enhanced version of BRET. It improves classical BRET by using NanoLuc luciferase, a smaller and more stable luciferase, which provides higher intensity luminescent intensity. NanoLuc emits light with a peak around 460 nm, which is ideal for efficient energy transfer to fluorescent acceptors with peak emissions in the 500–600 nm range, thus providing better spectral resolution and reducing interference from background signals. These advancements reduce background interference and enhance sensitivity, making NanoBRET ideal for HTS in drug discovery. BRET/NanoBRET is widely used to detect PPIs and to screen for small-molecule inhibitors, as it preserves the physiological relevance of cellular environments [31,32,33,34].

### 2.2. Bioluminescence Biosensors

**Figure 4:**
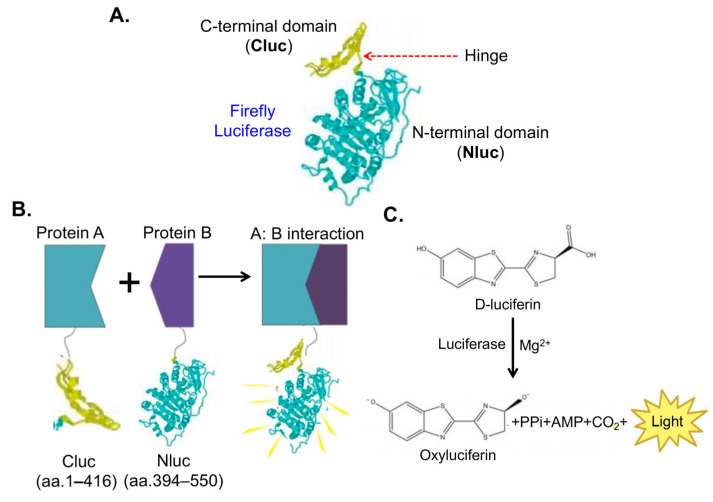
Schematic representation of bioluminescent biosensors using firefly SLCA monitoring PPIs. (**A**) Representation of the SLCA components in luciferase structure. (**B**) Split luciferase assays in monitoring PPIs. In this biosensor system, the firefly luciferase enzyme is split into two non-functional fragments: the N-terminal domain (Nluc, amino acids 1–416) and the C-terminal domain (Cluc, amino acids 394–550). These fragments are fused to two proteins of interest, Protein A and Protein B. When Protein A and Protein B interact, the two luciferase fragments are brought into close proximity, allowing them to reconstitute the functional enzyme. (**C**) Luciferase assays. In the presence of the substrate D-luciferin and cofactors such as Mg^2+^, the restored luciferase catalyzes the oxidation of D-luciferin to oxyluciferin, producing light. This luminescence signal indicates the interaction between the two proteins and can be quantified to measure the strength and dynamics of the PPIs.

Besides fluorescence-based biosensors, the bioluminescent biosensor is another type of biosensor commonly used in cancer drug discovery. Unlike fluorescence-based systems, bioluminescence does not require external light excitation, resulting in reduced background noise and enhanced sensitivity. Bioluminescence biosensors rely on light emission from biochemical reactions. The most common system uses luciferase enzymes, which catalyze substrate (e.g., luciferin) oxidation, producing photons as a byproduct. The two most widely used bioluminescence biosensor systems utilize firefly luciferase and NanoLuc luciferase. A SLCA system involves splitting the firefly luciferase into two non-functional segments: N-terminal (NLuc) and C-terminal (CLuc) luciferase (Figure 4A). These segments reconstitute into active firefly luciferase only when two proteins (Protein A and Protein B) of interest interact (Figure 4B,C). Researchers employ firefly biosensors to study signaling transduction and tumor growth in xenograft mouse models [13].

**Figure 5:**
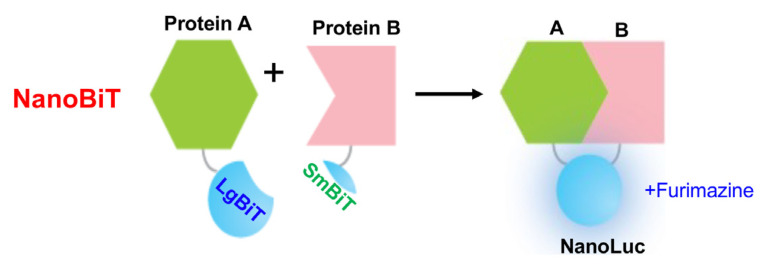
Schematic representation of the NanoBiT biosensors. The NanoBiT system utilizes two complementary luciferase fragments, LgBiT and SmBiT, which are fused to proteins of interest (Protein A and Protein B). Upon interaction between the two proteins, the fragments reassemble to form an active NanoLuc luciferase, generating bioluminescence in the presence of the substrate furimazine.

NanoLuc is a small bright luciferase enzyme that generates a robust light signal that is over 100 time stronger than firefly luciferases [35,36,37], making it ideal for detecting molecular interactions and cellular events in real time. Recently, a NanoBiT, a SLCA system using NanoLuc, was invented. In NanoBiT, NanoLuc is split into two non-functional halves, a large BiT fragment (LgBiT, 18 kDa) and a small BiT fragment (SmBiT, 1.3 kDa; 11 amino acids). These fragments are each fused to proteins of interest (e.g., Protein A and Protein B). When the target proteins interact, the LgBiT and SmBiT fragments come together to reconstitute a functional luciferase enzyme, which emits light upon the addition of the substrate (e.g., furimazine) (Figure 5). This luminescent signal is directly proportional to the interaction between the target proteins, allowing researchers to quantify real-time PPIs in living cells with high sensitivity [23,26].

Due to their high sensitivity and low background interference, bioluminescent biosensors are especially useful in in vitro applications. For instance, they can be used to track tumor growth, metastasis, or therapeutic responses in animal models by tagging cancer cells or specific proteins with bioluminescent markers [13,14,15,30,38,39,40]. The ability to monitor biological processes non-invasively over time makes bioluminescence biosensors a powerful tool in cancer research and drug discovery.

### 2.3. Software Tools and Data Analysis for Biosensors

Many software tools are generally used for data analysis for biosensors in HTS drug discovery. FRET/TR-FRET data analysis typically employs ratiometric calculations to determine energy transfer efficiency [41]. Software packages such as ImageJ 1.54 with the FRET Calculator plugin enable automatic analysis of FRET images, providing tools for background subtraction, photobleaching correction, and calculation of FRET efficiency [42]. Commercial platforms like LanthaScreen™ (Thermo Fisher, Burlington, ON, Canada) offer integrated solutions for TR-FRET data analysis, incorporating algorithms for time-resolved detection and signal normalization [43]. These tools typically calculate the ratio of acceptor to donor emission (e.g., 665 nm/615 nm for TR-FRET), applying corrections for signal overlap and background fluorescence.

For BRET, NanoBRET, and NanoBiT analyses, software platforms like GraphPad Prism 9 provide specialized tools for calculating BRET ratios and analyzing dose–response relationships [31]. These tools incorporate algorithms for (1) background subtraction and signal normalization; (2) the calculation of BRET ratios (acceptor emission/donor emission); (3) statistical analysis of signal significance; and (4) curve fitting for binding and kinetic studies. Promega’s Neo Instrument Software specifically designed for NanoBRET/NanoBiT assays offers automated data processing workflows, including data acquisition and control, kinetic analysis, quantitative analysis, data normalization and visualization, automation, and integrations with HTS.

HTS data analysis requires specialized software or platforms to handle large datasets [44,45]. Platforms such as Genedata Screener provides automated quality control, dose–response analysis, and hit identification [46]. Dotmatics Studies offer a data management platform that allows HTS data analysis along with assay data processing, visualization, and statistical analysis (https://www.dotmatics.com/). It can handle large datasets and integrates with other informatics systems, making it suitable for drug discovery workflows. The KNIME Analytics Platform enables custom workflow creation for automated data processing and analysis (https://www.knime.com/knime-analytics-platform) (accessed on 23 November 2024). These platforms incorporate algorithms for (1) Z-factor calculation for assay quality assessment; (2) hit identification and ranking; (3) dose–response curve fitting; and (4) false positive/negative filtering.

Significantly, while there are many reviews on biosensors [14,16,17,18,27,47,48,49,50] and cancer drug discovery [7,51,52,53,54,55,56], there is no comprehensive review on the use of fluorescence- and bioluminescence-based biosensors for cancer drug discovery, target validation, and signal transduction studies. In this review, we will discuss these aspects in greater detail.

## 3. Application of Fluorescence- and Bioluminescence-Based Biosensors in Drug Discovery Through HTS

While fluorescence- and bioluminescence-based biosensors have their respective advantages and limitations (Table 1), they are the most commonly used biosensors for HTS in drug discovery.

### 3.1. HTS Using Fluorescence-Based Biosensors

Fluorescence-based biosensors have become integral to HTS in drug discovery, particularly in cancer research. Techniques such as FRET, TR-FRET, BRET, and NanoBRET provide exceptional sensitivity for monitoring PPIs, kinase activities, and other cellular processes critical for cancer progression. These methods accelerate the identification of small-molecule inhibitors targeting key signaling pathways. Below, we examine various fluorescence-based biosensors and their applications in HTS (Table 2), drawing insights from recent studies.

#### 3.1.1. FRET Biosensors

FRET-based assays serve as powerful tools in HTS, providing real-time insights into molecular interactions and protein dynamics within live cells. By utilizing non-radiative energy transfer between two fluorophores, these assays detect subtle changes in molecular proximity (Figure 1), making them valuable across therapeutic areas, including cancer, neurodegenerative diseases, and inflammation.

Several studies demonstrate the versatility of FRET biosensors in drug screening (Table 2). He et al. (2019) employed FRET-based biosensors to measure ERK and AKT kinase activities in triple-negative breast cancer (TNBC) cells, revealing differential kinase dependencies and helping identify inhibitors to overcome resistance [57]. Similarly, Senarisoy et al. (2020) used FRET technology to study interactions between hNTH1 and YB1, crucial drivers of cancer progression, identifying molecules that sensitize tumors to chemotherapy [58]. Recent advancements have further enhanced FRET’s utility. Liu et al. (2021) integrated FRET with next-generation sequencing (NGS) to detect subtle kinase activities, such as ZAP70, in immune cells, improving biosensor sensitivity for HTS [59]. Hao et al. (2022) developed a peroxiredoxin-based FRET sensor for screening cancer therapeutics targeting H_2_O_2_-mediated pathways [60].

#### 3.1.2. TR-FRET Biosensors

Based on FRET assays, an advanced version called TR-FRET has been developed. TR-FRET assays build on conventional FRET by reducing background noise through time-gated measurements, improving signal stability and sensitivity (Figure 2). This method has proven effective in HTS across a range of biological applications (Table 2). Zhang et al. (2020) employed TR-FRET to discover inhibitors targeting the CBP bromodomain, disrupting oncogenic transcriptional activity and inhibiting MYC expression [61]. Loss of tumor suppressor genes by mutations is a hallmark of cancers [62]. Loss of function by mutations in SMAD4 contributes to the development of various types of cancers [63]. However, directly targeting mutant SMAD4 for cancer therapy remains challenging due to the lack of system screening for drugs reactivating mutant SMAD4 function. To address this challenge, Tang et al. developed a TR-FRET biosensor that captures the dynamic differential interaction between the SMAD4 and SMAD4R361H mutant with its functional binding partner SMAD3 [64]. Through HTS using this biosensor, they identified Ro-31-8220, a bisindolylmaleimide derivative, as a SMAD4R361H/SMAD3 interaction inducer. This compound reactivates dormant SMAD4R361H-mediated transcriptional activity and restores TGF-β-induced tumor suppression in SMAD4 mutant cancer cells [64]. Furthermore, TR-FRET has also been widely used to monitor PPIs. For example, Xiong et al. (2018) used TR-FRET to develop an ultra HTS (uHTS) platform for screening disruptors of NSD3-MYC interactions, identifying inhibitors that could block NSD2-MYC-induced oncogenic activation [65]. In addition, Yang et al. and Ouyang et al. developed a cell lysate-based TR-FRET assay to monitor MKK2-MYC and SMAD4-SMAD2 PPI, respectively, and identified a quinoline derivative SGI-1027 and gambogic/gambogenic acid as potent inhibitors for MKK2-MYC and SMAD4-SMD3 PPIs [28,29]. Moreover, Du et al. (2024) adapted TR-FRET for screening immune-related targets by designing a biosensor monitoring the SYK-FCER1G interaction. Their miniaturized 1536-well uHTS assay identified hematoxylin as a disruptor of this pathway, highlighting its potential for treating immune-related cancers [66].

TR-FRET applications extend to other signaling pathways. Singh et al. developed a TR-FRET demethylation screen assay for HTS and identified geldanamycin and its analog 17-DMAG as histone lysine demethylase (KDM) inhibitors, which inhibit tumor growth in alveolar rhabdomyosarcoma [67]. Lo et al. (2018) developed a TNFR TR-FRET biosensor and identified zafirlukast and triclabendazole as inhibitors of TNFR1-induced IκBα degradation and NF-κB activation [32]. After HTS in living cells, Larson et al. (2023) employed the method to identify modulators of the KRAS A146T mutant, a challenging target in oncology. They developed a novel high throughput TR-FRET assay leveraging the reduced nucleotide affinity of KRAS A146T. This assay detects small molecules that either allosterically modulate GDP affinity or directly compete with bound nucleotides. Through HTS of a diversity library containing over 83,000 compounds and subsequent validation, they identified UNC10104889 as a novel compound that inhibits KRAS GTPase activity, offering new therapeutic opportunities for colorectal and pancreatic cancers [68].

**Table 2:** Applications of fluorescence- and bioluminescence-based biosensors for HTS in cancer drug discovery.

Biosensor Type	Target/Pathway	Key Findings	Reference
FRET	ERK and AKT kinases	- Identified several kinase inhibitors differentially inhibit ERK and AKT	He et al. (2019) [57]
	hNTH1-YB1 PPI	- Identified SMs disrupting hNTH1-YB1 PPI - Identified 2 SMs sensitizing breast cancer cells to chemotherapeutic reagent cisplatin	Senarisoy et al. (2020) [58]
	ZAP70	- Established FRET-Seq method - Identified Sunitinib as repurposed drug	Liu et al. (2021) [59]
	H_2_O_2_-mediated pathways	- Established genetically encoded FRET probe for peroxiredoxin-2 (Prx2) oxidation - Identified an antifungal drug SMER3 as an oxidant-inducing drug	Hao et al. (2022) [60]
TR-FRET	CBP bromodomain	- Discovered inhibitors targeting CBP bromodomain - Inhibited MYC expression	Zhang et al. (2020) [61]
	SMAD4R361H-SMAD3	- Identified Ro-31-8220 as interaction inducer - Restored TGF-β-SMAD-induced tumor suppression	Tang et al. (2021) [64]
	NSD3-MYC PPI	- Identified TF-3 as an inhibitor of NSD3-MYC PPI	Xiong et al. (2018) [65]
	MKK3-MYC PPI	- Identified a quinoline derivative SGI-1027 as a potent inhibitor of MKK3-MYC PPI	Yang et al. (2021) [29]
	SMAD4-SMAD3 PPI	- Identified gambogic acid and gambogenic acid as compounds disrupting SMAD4-SMAD3 PPI	Ouyang et al. (2024) [28]
	SYK-FCER1G	- Identified hematoxylin as an inhibitor of SYK-FCER1G PPI	Du et al. (2024) [66]
	KDM	- Identified geldanamycin and its analog 17-DMAG as KDM inhibitors.	Singh et al. (2020) [67]
	KRAS GTPase	- Identified UNC10104889 as an inhibitor of KRAS A146T	Larson et al. (2023) [68]
BRET/ NanoBRET	RAS-RAF PPI	- Identified several compounds including Ophiobolin and NSC145366 as inhibitors of RAS-RAF signaling	Durrant et al. (2021) [33]
	PTK7-β-catinin	- Identified several compounds (01065, 03653, and 20279) as inhibitors PTK7-β-catinin in colon cancer	Ganier et al. (2022) [69]
NanoBiT	RAF	- Establish a biosensor detecting RAF dimerization - Identified several RAF inhibitors (e.g., TAK532) promoting RAF dimerization	Miyamoto et al. (2019) [70]
	PP1 and PP2A holoenzymes	- Identify SM 18R1K7 that inhibit PP1 by disrupting PP1-MYPT1 PPI	Claes and Bollen (2023) [71]
	YAP/TAZ-TEAD	- Identified Celastrol SM disrupting YAP/TAZ-TEAD PPI and inhibiting cancer cell proliferation	Nouri et al. (2019) [24]
	RAS-effector PPI	- Establish HTS for examing PPI of RAS and its effectors (e.g., PI3K)	Cooley et al. (2020) [72]
HiBiT	PD-L1	- Identified SMs regulating PD-L1 stability - Advanced immunotherapy using new SMs	Uchida et al. (2021) [73]
	YAP/TAZ	- Identifed many novel SMs (e.g., Avanafi, β-catenin, Fluvastatin) causing YAP/TAZ degradation	Wu et al. (2023) [40]

While TR-FRET is a powerful tool, challenges persist in assay design, including donor–acceptor pair optimization and fluorophore stability. However, innovations such as machine learning for data analysis continue to improve assay reliability and scalability, as reported by Shimizu et al. (2021) [74].

#### 3.1.3. BRET and NanoBRET Biosensors

Besides FRET/TR-FRET-based biosensors, BRET and its advanced version NanoBRET (Figure 3) have become pivotal in HTS (Table 2), offering unparalleled sensitivity for studying PPIs in live cells [31]. NanoBRET has proven particularly valuable in monitoring the RAS-RAF interaction, which is critical in cancer progression, identifying both inhibitors and pathway-specific modulators [33]. Researchers have successfully applied this technology to HTS for drugs targeting the PTK7-β-catenin interaction, a key driver in colorectal cancer [69].

### 3.2. HTS Using Bioluminescent NanoBiT Biosensors

Besides fluorescence-based biosensors, bioluminescence-based biosensors are widely used for HTS (Table 2). The most sensitive bioluminescent biosensor used for HTS is NanoBiT. NanoBiT has emerged as a cutting-edge bioluminescent tool for drug discovery, specifically designed for monitoring protein levels and PPIs in cells (Figure 5; Table 2). The small size of its fragments and the high sensitivity of the NanoLuc system make NanoBiT particularly suitable for dynamic real-time applications across diverse drug discovery platforms (Table 1). Importantly, NanoBiT retains high specificity and sensitivity even in the presence of widely used kinase inhibitors. This resilience proves critical for screening chemical libraries, where assay interference from certain compounds might lead to false-positive or false-negative results [75].

Researchers have successfully employed NanoBiT biosensors for screening drugs disrupting PPIs (Table 2) [76]. For example, Miyamoto et al. (2019) developed NanoBiT-based biosensors to detect RAF dimerization, a process contributing to resistance in RAF kinase-targeted cancer therapies [70]. Their study demonstrated that split luciferase complementation assays can be used for the HTS of drug candidates, identifying inhibitors that specifically modulate RAF dimerization. Similarly, Claes and Bollen (2023) showed NanoBiT’s utility in screening SMs that modulate phosphatase subunit interactions [71]. Their SLCA provided a robust platform for the HTS of compounds that interfere with PP1 phosphatase holoenzyme formation, which has therapeutic implications for diseases such as cancer and neurodegenerative disorders. Recent studies suggest that YAP-TEAD PPI is critical for tumorigenesis [77]. Therefore, targeting YAP-TEAD PPI is a very promising therapeutic strategy for cancer. However, no drug targeting YAP-TEAD has been approved by the FDA. To screen for new SMs disrupting YAP-TEAD PPI, we recently developed an ultra-bright NanoLuc biosensor to quantify YAP/TAZ-TEAD PPIs both in living cells and in vitro using biosensor fusion proteins purified from bacteria [24]. Through in vitro HTS using this biosensor protein purified from bacteria, we identified and validated Celastrol as a novel inhibitor of the YAP/TAZ-TEAD interaction. Further studies demonstrated that Celastrol can inhibit cancer cell proliferation, transformation, and migration by disrupting the YAP/TAZ-TEAD interaction [24]. Using a similar cell-free approach, Cooley et al. (2020) developed a NanoBiT biosensor to detect weak protein interactions between RAS and its effectors [72]. This method enabled the screening of poorly soluble protein domains, demonstrating NanoBiT’s flexibility in challenging drug discovery applications. Such versatility has made NanoBiT a valuable asset for identifying inhibitors that target PPIs relevant to cancer and other diseases.

Targeted protein degradation (TPD) is a promising therapeutic strategy that involves the selective destruction of disease-related proteins [78,79]. NanoBiT technology has been adapted to a HiBiT system for use in TPD studies, providing a sensitive and high-throughput platform for monitoring protein levels in real time. In this HiBiT system, the 11-amino acid SmBiT sequence is modified to HiBiT with the same size but has a much higher affinity compared to LgBiT, another component of the NanoBiT system (Figure 5). When HiBiT is fused to a protein of interest and expressed in cells, it can spontaneously associate with the LgBiT fragment, forming a functional luciferase enzyme. This reconstituted enzyme emits light in the presence of a substrate, allowing for the quantification of HiBiT-tagged protein levels. Moreover, Lankford et al. (2024) developed a protocol for HiBiT tagging endogenous proteins using CRISPR-Cas9, which enhances the ability to study endogenous proteins in real time, which is essential for understanding the effects of drug candidates on physiologically relevant protein targets [80].

The HiBiT system is particularly useful for HTS for SMs causing protein degradation in living cells due to its high sensitivity and simplicity [81]. For example, Uchida et al. (2021) used HiBiT-tagged PD-L1 proteins to screen chemical libraries for compounds that modulate PD-L1 expression [73]. This approach identified several compounds that upregulate or downregulate PD-L1, a key immune checkpoint molecule. Modulating PD-L1 expression is central to the development of immune checkpoint inhibitors, a class of drugs that has revolutionized cancer immunotherapy. In addition, we recently utilized HiBiT biosensors to monitor the stability of YAP/TAZ proteins in breast cancer cells [40]. Our HTS identified many novel SMs causing the degradation of oncogenic YAP/TAZ proteins, providing a valuable tool for developing YAP/TAZ-targeted TPD anti-cancer therapeutics. Moreover, Lin et al. (2024) introduced lysine-deficient HiBiT and NanoLuc variants to eliminate potential degradation artifacts caused by traditional tagging systems [82]. Their study highlighted that these variants maintain the sensitivity and specificity of the original NanoBiT system, making it an ideal tool for studying the effects of protein degraders like PROTACs.

## 4. Application of Biosensors in Drug Validation

Biosensors have revolutionized drug discovery by enabling real-time sensitive monitoring of biological processes, providing critical insights into the efficacy, target engagement, and mechanism of action of drug candidates. In drug validation, various biosensors—including FRET, NanoBRET, and NanoBiT—play pivotal roles in assessing SM drugs, including PROTACs. This section discusses the roles of these biosensors in drug validation.

### 4.1. FRET and TR-FRET Biosensors

FRET biosensors are widely used to investigate PPIs and protein conformational dynamics upon ligand binding. These assays prove crucial in validating drug efficacy by tracking how SMs modulate these interactions. For example, Sahin et al. (2021) applied FRET biosensors to validate novel inhibitors disrupting anti-apoptotic BCL-2 complexes targeting the apoptotic pathway in cancer [83]. Similarly, Borysko et al. (2018) utilized FRET to visualize the drug-induced dissociation of BRD4 from its interaction partners, contributing to the identification of novel BRD4 inhibitors [84]. FRET biosensors demonstrate particular effectiveness in live-cell assays, facilitating the study of drug effects in physiological conditions. Farmer et al. (2022) used FRET-based peptide biosensors to monitor G protein-coupled receptors (GPCR) activation and quantify the influence of intracellular allosteric modulators, expanding FRET applications in GPCR-targeting drug validation [85].

TR-FRET builds on FRET principles by using time-resolved detection to minimize background fluorescence (Figure 2). This enhanced sensitivity makes TR-FRET particularly valuable in studying binding affinities and protein conformational changes in drug validation. Lin et al. (2021) developed a TR-FRET assay to quantify ternary complex formation between BRD4, PROTAC molecules, and CRBN ligase, providing insights into the PROTAC mechanism of action [86]. In addition, Ali Abed et al. (2023) applied TR-FRET assays to evaluate novel inhibitors disrupting the Keap1-Nrf2 interaction, a key target for oxidative stress modulation [87]. Moreover, Payne et al. (2023) further demonstrated the versatility of TR-FRET by using it to quantify endogenous BRD4 protein levels in cancer cells, enabling rapid validation of small-molecule BRD4 degraders [88]. TR-FRET’s adaptability makes it a valuable tool for validating a wide range of therapeutic candidates beyond PROTACs.

### 4.2. BRET and NanoBRET Biosensors

BRET and NanoBRET biosensors provide a non-invasive highly sensitive platform for studying molecular interactions in living cells (Figure 3). These techniques leverage energy transfer from luciferase to a fluorophore, offering real-time quantitative insights into drug–target engagement. NanoBRET, with its improved luminescence and reduced background noise, has become particularly valuable in drug validation.

#### 4.2.1. Ligand–Receptor Binding Inhibitor Validation

NanoBRET assays have been widely applied to study ligand–receptor interactions. Kozielewicz et al. (2022) used NanoBRET to evaluate the binding affinities of SMs targeting the SMO receptor in Hedgehog signaling, providing critical insights into competition with known agonists [89]. Similarly, Lay et al. (2022) developed a NanoBRET assay to study the intracellular binding kinetics of BET inhibitors, helping optimize drug efficacy by measuring dissociation rates [90].

#### 4.2.2. Kinase Inhibitor Validation

NanoBRET has increasingly proven valuable for studying kinase inhibitors. The polo-like kinases (PLKs) are serine/threonine (Ser/Thr) protein kinases that play key roles in the cell cycle and mitosis and are often dysregulated in cancer [91]. Although many PLK inhibitors have been developed [91], there is no system to accurately evaluate the cellular potency of these inhibitors. Yang et al. (2023) developed cell-permeable NanoBRET probes to monitor PLK1 engagement [92]. They have shown that live cell NanoBRET target engagement assays can be used to measure PLK1 inhibitor binding kinetics, providing critical insights into kinase inhibitor cellular potency in real time and useful information on improved validation of PLK1-targeting therapeutics. Moreover, Kong et al. (2024) used the NanoBRET biosensor to validate inhibitors targeting MERTK and AXL kinases, which are essential in overcoming chemoresistance in lung cancer cells [93].

#### 4.2.3. PROTAC and Molecular Glue Validation

NanoBRET has become instrumental in PROTAC validation, offering valuable insights into their intracellular target engagement, bioavailability, and mechanisms of action. By measuring real-time interactions between PROTACs, target proteins, and E3 ligases, NanoBRET enables quantitative analysis of these interactions in living cells. This approach provides detailed data on ternary complex formation, intracellular accumulation, and target degradation, which is essential for optimizing PROTAC design. One of the primary applications of NanoBRET in PROTAC validation involves assessing target engagement within live cells. The technology enables measurement of the binding affinity between PROTACs and their targets by monitoring the energy transfer between NanoLuc-tagged proteins and fluorescent tracers. For instance, Vasta et al. (2021) presented a high-throughput NanoBRET-based assay to evaluate the intracellular permeability and target engagement of PROTACs targeting CRBN and VHL E3 ligases [94]. This approach helps prioritize PROTAC candidates based on their relative intracellular availability and engagement with E3 ligases, making NanoBRET a critical tool for drug discovery.

Beyond target engagement, NanoBRET plays a significant role in understanding the ubiquitination process, a key step in PROTAC-induced protein degradation. Bai et al. (2022) demonstrated how NanoBRET assays can model and predict target protein ubiquitination efficiency [95]. These insights prove crucial for evaluating whether ternary complex formation leads to productive ubiquitination and subsequent proteasomal degradation. The ability of NanoBRET to measure such interactions in a live-cell context provides a comprehensive view of how PROTACs induce degradation, aiding in the optimization of their design.

NanoBRET also facilitates the exploration of new E3 ligase ligands in PROTAC design. Pei et al. (2023) used NanoBRET to confirm that a Piperlongumine-based PROTAC recruits KEAP1 as its E3 ligase to degrade CDK9 [96]. This approach expands the toolkit for PROTAC design by identifying novel E3 ligases for targeted degradation, thus broadening applications across different cellular contexts and protein targets. Furthermore, NanoBRET helps assess the bioavailability and intracellular accumulation of PROTACs, which are critical parameters influencing drug efficacy. Yu et al. (2023) developed a NanoBRET-based platform to measure the intracellular accumulation of PROTACs, providing a quantitative method to evaluate their cellular permeability [97]. This information is essential for improving the pharmacokinetics of PROTACs and ensuring that they reach sufficient intracellular concentrations to induce target degradation effectively. Zerfas et al. (2023) further advanced the application of NanoBRET in drug validation by developing a CRBN-specific NanoBRET assay [98]. This assay measures the occupancy of the CRBN binding site, allowing researchers to study the relationship between CRBN engagement and target degradation. Such data are vital for optimizing PROTACs that rely on CRBN-mediated degradation pathways, providing a detailed understanding of the interaction dynamics that drive their efficacy.

#### 4.2.4. Covalent Inhibitor Validation

Covalent inhibitors, designed to form irreversible bonds with target proteins, benefit from NanoBRET’s ability to monitor real-time interactions and verify the specificity of drug binding. In their 2022 study, Borsari et al. used NanoBRET to confirm covalent binding of phosphoinositide 3-kinase α (PI3Kα) inhibitors to a distal cysteine residue, demonstrating how this method enables precise assessment of target engagement and off-target interactions in live cells [99]. By combining NanoBRET with X-ray crystallography and mass spectrometry, the authors validated the covalent interaction of acrylamide-based inhibitors with PI3Kα, providing a comprehensive view of drug action. In addition, Weeks et al. (2022) also demonstrated the utility of NanoBRET biosensors in live-cell validation of Ras covalent inhibitors. Their study leveraged a Ras activity biosensor to track the inhibition kinetics of KRasG12C inhibitors in living cells [100]. This approach enabled real-time observation of Ras activity modulation, thus facilitating the validation of covalent inhibitors and their dynamic effects within a cellular environment. Together, these studies underscore the effectiveness of using NanoBRET in validating covalent drug mechanisms and refining therapeutic strategies.

#### 4.2.5. Validation of Candidate Inhibitors from DEL Screening

DEL screening represents a high-throughput technology for identifying SMs that bind to target proteins or other biological macromolecules. The method creates large libraries of SMs, each attached to a unique DNA barcode identifier. This DNA tag encodes the identity of the SM and allows for the rapid screening of millions to billions of compounds in a single experiment [101]. NanoBRET biosensors are increasingly used to validate SMs identified from DEL screening. Teske et al. (2023) developed cell-permeable BRET probes from DEL hits targeting aurora kinase A, allowing real-time assessment of target engagement [34]. In addition, Madasu et al. (2024) extended this approach to EPH receptor kinase inhibitors, using NanoBRET to evaluate cellular selectivity and potency, guiding further optimization [102]. These studies suggest that NanoBRET can be used for the validation of hits from DEL screening.

### 4.3. NanoBiT Biosensors

NanoBiT biosensors have become an essential tool in drug validation, particularly for monitoring real-time PPI in cellular processes. For instance, Hinz et al. (2021) developed a NanoBiT assay to monitor the interaction between human Geranylgeranyltransferase Type I (GGTase I) and its substrate Rap1B, providing real-time insights into interaction dynamics [103]. This sensitive platform is instrumental for screening and the validation of GGTase I inhibitors, potential therapeutic agents in cancer treatment. NanoBiT effectively captures subtle changes in protein interactions caused by drug candidates, facilitating the assessment of drug efficacy in modulating these interactions. Similarly, Reyes-Alcaraz et al. (2022) designed a NanoBiT-based assay to quantify membrane protein internalization and recycling, a process critical to both physiological and pathological mechanisms. This assay is particularly valuable for validating drug candidates targeting membrane proteins, especially in the context of drug–receptor interactions [21].

Moreover, NanoBiT has been applied in diverse areas such as receptor oligomerization, as demonstrated by Morató et al. (2023), where it was used to study the heterodimerization of S1R with the binding immunoglobulin protein (BiP) [104]. This application highlights NanoBiT’s utility in validating compounds that affect PPIs, proving its relevance in drug discovery for cancer and neurodegenerative diseases. The versatility of NanoBiT extends to the therapeutic drug monitoring (TDM) of monoclonal antibodies, as shown by Campbell et al. (2023) [105], enabling real-time monitoring of drug levels to improve treatment outcomes. Additionally, Claes and Bollen (2023) employed NanoBiT for the validation of phosphatase subunit modulators [71], while Rohrer et al. (2023) utilized NanoBiT to investigate RAF kinase dimerization, further showcasing its value in drug validation for key signaling pathways like RAF/MEK/ERK [106].

Lastly, we developed a NanoBiT biosensor for GSDMD, a critical effector in pyroptosis linked to cancer and inflammation-related diseases [107]. This biosensor allowed for the quantification of GSDMD’s intramolecular interaction and levels both in vitro and in vivo, exemplifying NanoBiT’s potential in cancer research and therapeutic validation.

## 5. Biosensors in Studying Cancer Cell Signaling Pathways

Advances in biosensor technologies significantly enhanced our understanding of cancer cell signaling pathways by enabling the study of PPIs, ligand binding, enzyme activities, protein levels, and conformational changes. These biosensors provide insights both in vitro and in real time within living cells or in vivo cancer xenograft mouse models. This section discusses various biosensor technologies, such as NanoBRET, NanoBiT, and FRET, and their applications in uncovering key aspects of cancer signaling pathways.

### 5.1. FRET-Based Biosensors

FRET-based biosensors provide valuable insights into kinase activities and protein interactions within specific cellular compartments. These biosensors leverage energy transfer between fluorophores to track molecular interactions with high temporal resolution. Hsu et al. (2014) applied a split-luciferase complementation assay to identify multiple kinases involved in the assembly of ion channel complexes [108]. Ouyang et al. (2024) [28] further demonstrated FRET biosensors’ capabilities by developing a tool to monitor C-terminal Src kinase (CSK) activity in live cells, tracking the kinase’s activity within distinct membrane regions. This approach highlights how FRET biosensors can provide insights into the spatial dynamics of signaling molecules.

### 5.2. NanoBRET Biosensors

NanoBRET has emerged as a powerful tool for studying ligand binding and protein interactions with high spatiotemporal resolution. This technology enables researchers to monitor drug–target engagement both in live cells and in vitro. Alcobia et al. (2018) used NanoBRET to visualize ligand binding to β2-adrenoceptors in real time, demonstrating the method’s effectiveness in providing dynamic insights into receptor–ligand interactions [109]. Stoddart et al. (2015) further improved ligand binding assays using NanoBRET to monitor GPCR activity in real time, surpassing traditional radioligand binding techniques in sensitivity [110]. Additionally, NanoBRET has provided insights into neo-protein interactions in cancers, revealing vulnerabilities associated with oncogenic mutations, such as BRAF V600E [111].

Dosquet et al. (2021) showcased the NanoBRET’s versatility by developing biosensors to monitor receptor tyrosine kinase (RTK) activity, focusing on EGFR and AXL [112]. Building on this, Boon et al. (2023) developed REGA-SIGN, a suite of NanoBRET-based biosensors capable of tracking G protein activation across multiple G protein families, illustrating the broad applicability of NanoBRET in signaling pathway studies [113].

### 5.3. Firefly Luciferase Biosensor and NanoBiT Biosensors

Firefly luciferase biosensors and NanoBiT biosensors are widely employed to study protein interactions and pathway regulation due to their ability to monitor real-time interactions with high sensitivity. LATS kinase is a tumor suppressor and core component of the Hippo pathway that plays critical roles in various cellular functions such as cell cycle regulation and apoptosis [38,114,115]. However, the upstream signaling pathway regulating LATS function is largely unknown and there is no system to monitor LATS kinase activity in living cells in real time. To address these issues, we recently developed a bioluminescence-based biosensor using firefly split luciferase assays to monitor the activity of LATS kinase, a core component of the Hippo signaling pathway. This LATS biosensor (LATS-BS) enabled non-invasive real-time measurement of LATS activity in vitro and in vivo with high sensitivity and quantification capabilities [116]. Using the LATS-BS and a library of kinase inhibitors, we conducted a screen to identify kinases modulating LATS activity. This screen revealed VEGFR as an upstream regulator of the Hippo signaling pathway. We found that VEGFR activation by VEGF triggers PI3K/MAPK signaling, which subsequently inhibits LATS and activates the Hippo effectors YAP (Yes-associated protein) and TAZ (transcriptional coactivator with PDZ-binding motif). Further experiments showed that the Hippo pathway is a critical mediator of VEGF-induced angiogenesis and tumor vasculogenic mimicry. The inhibition of YAP/TAZ reduced VEGF-stimulated angiogenesis in multiple in vitro and in vivo models [38]. We have also developed more sensitive NanoBiT biosensors for monitoring LATS activity [116]. Gain-of-functional and loss-of-functional screenings using this LATS biosensor identified many receptor tyrosine kinases (e.g., ALK, FGFR, AXL, MERTK, and RET) as novel regulators of the Hippo signaling pathway in tumorigenesis, metastasis, and immune evasion [116,117]. By using this biosensor for a gain-of-functional screening, we have also identified several tyrosine phosphatases including PTPN12 as a novel regulator of LATS kinase and the Hippo pathway [118]. Our studies strongly suggest that biosensors can be a very powerful tool to monitor the activity of a kinase for screening new therapeutic targets for cancer. Similarly, Poti et al. (2023) further expanded NanoBiT applications with PhALC (Phosphorylation-Assisted Luciferase Complementation) [119], enabling real-time monitoring of kinase activity and PPIs. In addition, Kupcho et al. (2019) developed a real-time bioluminescent annexin V assay using NanoBiT to detect apoptosis [120], while Inoue et al. (2019) applied this technology to comprehensively profile GPCR-G protein coupling selectivity [121]. Moreover, Zeghal et al. (2023) and Pipchuk et al. (2024) demonstrated the utility of NanoBiT biosensors in studying GPCR signaling and Merlin tumor suppressor protein conformation, respectively [22,122].

## 6. Challenges and Future Perspectives

The application of biosensors in drug discovery, particularly in cancer research, presents several significant challenges. Despite their precision and ability to monitor real-time molecular interactions, the integration of biosensors into HTS for drug compounds faces technical and operational hurdles. A primary challenge lies in designing biosensors that accurately mimic the physiological conditions of cancer cells. This includes ensuring biosensors can function effectively in complex environments, such as 3D tumor models, where traditional 2D cultures fail to replicate the intricate interactions of the tumor microenvironment. The scalability of biosensors for HTS presents another significant challenge. The sensitivity of biosensors often requires sophisticated instrumentation and precise calibration, limiting their accessibility for large-scale drug screening. Additionally, biosensors sometimes exhibit limited stability over long periods, complicating their use in extended screening processes. Achieving high specificity while avoiding cross-reactivity between biomolecules remains challenging, as unintended interactions can lead to false positives, reducing result reliability. Moreover, validating hits from HTS using biosensors can also be challenging, requiring multiple approaches to exclude off-target hits obtained due to quenching of bioluminescent or fluorescent signals.

Looking ahead, the future of biosensors in drug discovery appears promising. Advances in nanomaterials, such as carbon nanotubes and quantum dots, offer the potential to enhance biosensor sensitivity and specificity. These materials can improve signal transduction and lower detection limits, making biosensors more efficient for early disease detection and personalized medicine. Additionally, integrating AI with biosensor technologies could help analyze large datasets generated by HTS, facilitating the identification of novel drug candidates and optimizing biosensor design.

## 7. Conclusions

Biosensors such as FRET, TR-FRET, NanoBRET, and NanoBiT have significantly advanced our understanding of cancer cell signaling pathways. These technologies enable real-time, sensitive, and specific detection of protein levels, PPI, ligand binding, and enzyme activities in live cells. By uncovering novel regulatory mechanisms, identifying new drug targets, and providing valuable tools for drug discovery and validation, biosensors play an essential role in modern cancer research and drug discovery. As these technologies continue to evolve, they promise to yield deeper insights into the complex signaling networks that underlie cancer biology, facilitating the development of innovative therapeutic strategies.

## Figures and Tables

**Table 1:** Comparison of different types of biosensors.

Biosensors	Advantages	Limitations
FRET/TR-FRET	High spatial resolution (<10 nm) Real-time measurements in living cells Multiple fluorophore pairs available Well-suited for high-throughput screening	Potential photobleaching and autofluorescence Requires careful controls for spectral overlap Expression level dependence Limited tissue penetration
BRET/NanoBRET	No external excitation needed, reducing background Enhanced sensitivity with NanoLuc Better tissue penetration for in vivo imaging Good signal-to-noise ratio	Requires substrate addition Limited multiplexing options Size of luciferase tag may affect protein function
NanoBiT	Small tag size (11 amino acid SmBiT) Extremely bright signal Good for protein stability/levels studies Works well in cell-free systems	Irreversible complementation Requires close proximity Limited to binary interactions

## Abbreviations

2D	Two-dimensional
AI	Artificial intelligence
BiP	Binding immunoglobulin protein
BRET	Bioluminescence resonance energy transfer
CFP	Cyan fluorescent protein
CLuc	C-terminal luciferase
CRISPR	Clustered regularly interspaced short palindromic repeats
CSK	C-terminal Src kinase
CTC	Circulating tumor cells
ctDNA	Circulating tumor DNA
DEL	DNA encoded library
EGFR	Epithelial growth factor receptor
Eu	Europium
FDA	Federal drug administration
FP	Fluorescence polarization
FRET	Fluorescent resonance energy transfer
GFP	Green fluorescent protein
GGTase I	Geranylgeranyltransferase Type I
GPCR	G protein-coupled receptors
HTS	High throughput screening
ITC	Isothermal titration calorimetry
KDM	Histone lysine demethylase
LAST-BS	LATS biosensor
LgBiT	Large BiT
NanoBiT	NanoLuc binary technology
NanoBRET	NanoLuc BRET
NGS	Next generation sequencing
NLuc	N-terminal luciferase
NSCLC	None-small cell lung cancer
PhALC	Phosphorylation-assisted luciferase complementation
PI3K α	Phosphoinositide 3-kinase α
PLK	Polo-like kinases
PPI	Protein–protein interaction
PROTAC	Proteolysis targeting chimera
Prx2	Peroxiredoxin-2
RLuc	Renilla luciferase
Ser	Serine
SLCA	Split-luciferase complementation assays
SM	Small molecule
SmBiT	Small BiT
SPR	Surface plasmon resonance
TAZ	Transcriptional coactivator with PDZ-binding motif
Thr	Threonine
TNBC	Triple negative breast cancer
TPD	Targeted protein degradation
TR-FRET	Time-lapse FRET
uHTS	Ultra HTS
YAP	Yes-associated protein
YFP	Yellow fluorescent protein
